# Intelligent Vehicle Path Tracking Control Method Based on Curvature Optimisation

**DOI:** 10.3390/s23104719

**Published:** 2023-05-12

**Authors:** Qing Ye, Chaojun Gao, Yao Zhang, Zeyu Sun, Ruochen Wang, Long Chen

**Affiliations:** Automotive Engineering Research Institute, Jiangsu University, Zhenjiang 212013, China

**Keywords:** intelligent vehicle, path tracking, fuzzy sliding mode control, curvature optimisation, body stability

## Abstract

In this study, an intelligent vehicle (IV) path tracking control method based on curvature optimisation is proposed to reduce the comprehensive performance conflict of the system. This system conflict is caused by the mutual restriction between the path tracking accuracy and the body stability during the movement of the intelligent automobile. First, the working principle of the new IV path tracking control algorithm is briefly introduced. Then, a three-degrees-of-freedom vehicle dynamics model and a preview error model considering vehicle roll are established. In addition, a path tracking control method based on curvature optimisation is designed to solve the deterioration of vehicle stability even when the path tracking accuracy of the IV is improved. Finally, the effectiveness of the IV path tracking control system is validated through simulations and the Hardware in the Loop (HIL) test with various conditions forms. Results clearly show that the optimisation amplitude of the IV lateral deviation is up to 84.10%, and the stability is improved by approximately 2% under the *v_x_* = 10 m/s and *ρ* = 0.15 m^−1^ condition; the optimisation amplitude of the lateral deviation is up to 66.80%, and the stability is improved by approximately 4% under the *v_x_* = 10 m/s and *ρ* = 0.2 m^−1^ condition; the body stability is improved by 20–30% under the *v_x_* = 15 m/s and *ρ* = 0.15 m^−1^ condition, and the boundary conditions of body stability are triggered. The curvature optimisation controller can effectively improve the tracking accuracy of the fuzzy sliding mode controller. The body stability constraint can also ensure the smooth running of the vehicle in the optimisation process.

## 1. Introduction

As the three key technologies of intelligent vehicles (IVs), environmental perception, behavioural decision-making and path planning, and motion control have made great progress. Motion control obtains the desired path and speed information from the path planning module. Then, it gives instructions to the actuators, such as acceleration, braking, and steering, according to the current state of the vehicle and the surrounding environment information. Thus, it directly affects the driving safety, handling stability, and ride comfort of the vehicle. However, path tracking control systems still face great challenges in extremely complex real application scenarios [1].

Many scholars and researchers have conducted in-depth research on the path tracking technology of IV. N. A. Spielberg et al. [2] proposed a high-performance neural network vehicle model for autonomous driving. This model can learn the dynamic behaviour of a vehicle on a range of different surfaces by developing a two-layer feed-forward neural network, greatly improving the vehicle’s tracking accuracy under friction limit conditions. Reference [3] employed roll angle compensation to reduce steady-state errors. Then, a yaw rate reference for path tracking was generated. S. Cheng designed a path tracking controller for autonomous vehicles based on parameter uncertainty and speed variation to ensure the accuracy and robustness of trajectory tracking [4]. N. Awad [5] presented a model predictive control (MPC) for autonomous vehicle path tracking based on fuzzy logic switching. In [6], a constrained linear time-varying MPC was used to optimise vehicle lateral trajectory and improve path tracking accuracy. 

The following are observed from the above research: (1) The current research on IV motion control mainly focuses on improving tracking accuracy. The changes in body stability during the tracking process are ignored. (2) Precision optimisation is usually achieved by improving the control algorithm. A few optimisation analyses are performed from the perspective of the control model. Given the limitation of design ideas, the existing path tracking control algorithms meet the accuracy requirements in the actual driving process but neglect the tracking accuracy and body stability under extreme conditions, such as high speed and large curvature. Thus, further research on tracking control is required.

Many studies have incorporated body stability analysis into the path tracking process. M. Corno et al. [7] proposed a linear parameter-varying multiple-input single-output (LPV-MISO) H-infinity controller to ensure the effectiveness of an IV path tracking control system in aggressive operating conditions whilst ensuring passenger comfort. Y. Cai et al. [8] presented a hybrid control strategy for IV path tracking. This strategy uses PID control in the low-speed mode and MPC in the high-speed mode. It also determines the path tracking control mode through vehicle speed and then designs the control mode switching mechanism with stability supervision. L. Tang et al. [9] designed a cascaded MPC-PID controller. The expected output is the yaw rate. The PID controller was used to determine the reference yaw rate. A roll angle compensator was built to correct the motion model prediction and optimise the road curvature disturbance in the MPC control process. Z. Zhao et al. [10] proposed a path tracking controller with adaptive optimisation of preview distance. This controller solves the problems of driving comfort and body stability under a high speed and a small turning radius. Y. Chen et al. [11] designed a multi-constraint path tracking controller and a stability controller to generate the steering angles of the front and rear wheels, respectively. These controllers improve the path tracking ability and handling stability of autonomous vehicles under extreme conditions. The longitudinal and lateral coordinated stability control method proposed by L. Chen [12] can improve the transient response of unmanned vehicles under extreme conditions. It can also improve the trajectory tracking accuracy of unmanned vehicles and lateral stability during curve movement.

In summary, the current research mainly uses precision optimisation algorithms to improve all-around performance. However, realising the safe driving of the vehicle without deviation remains impossible in extremely complex real application scenarios. Moreover, improving the algorithm complicates the controller structure design. The process is too long, the feasibility is greatly reduced, and the practicality is weak. Therefore, the path tracking control system designed in the present study considers the changes in tracking accuracy and stability during the tracking process to solve the above problems. The coupling relationship between the two is analysed, and a path tracking controller based on curvature optimisation is proposed. Moreover, this study analyses the relationship between the desired path curvature and the tracking accuracy. The tracking accuracy is also improved whilst ensuring the stability of the vehicle body by optimising the curvature.

In Section 2, the path tracking controller based on curvature optimisation is introduced. The trajectory tracking control algorithm at different levels is established in Section 3. In Section 4, the simulation results of trajectory tracking controllers with and without subsystems are compared to verify the influence of each subsystem on the accuracy of the trajectory tracking control system. The conclusions are drawn in Section 6.

## 2. Path Tracing Control Framework

**Figure 1:**
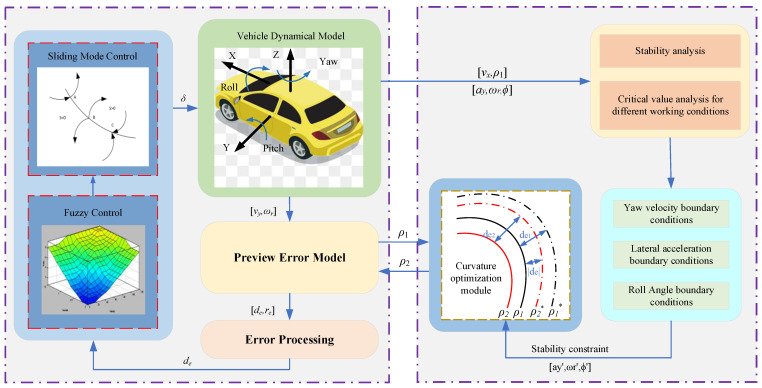
Path tracking control architecture diagram.

The framework of the IV path tracking controller based on curvature optimisation consists of two parts: a path tracking control system and curvature optimisation system. The path tracking control system includes a dynamic model, preview error model, and fuzzy sliding mode controller. The curvature optimisation system consists of a path tracking control system, stability analysis module, stability boundary condition determination module, and curvature optimisation unit. The path tracking control framework diagram is shown in Figure 1.

## 3. Design of Path Tracking Controller Based on Curvature Optimisation

### 3.1. Design of the Path Tracking Control System 

The path tracking control system (shown in Figure 1) consists of a dynamic model, a preview error model, and a fuzzy sliding mode controller. The dynamic model outputs the real-time vehicle state parameters required by the preview error model. Then, the vehicle state parameters are input into the preview error model to obtain the lateral deviation and the directional deviation. Furthermore, the processed error data are input into the fuzzy sliding mode controller to obtain the desired front wheel angle. Finally, the angle is input into the dynamic model again to complete the closed-loop control of the lateral motion and realise the path tracking of the IV.

#### 3.1.1. Kinetic Model

**Figure 2:**
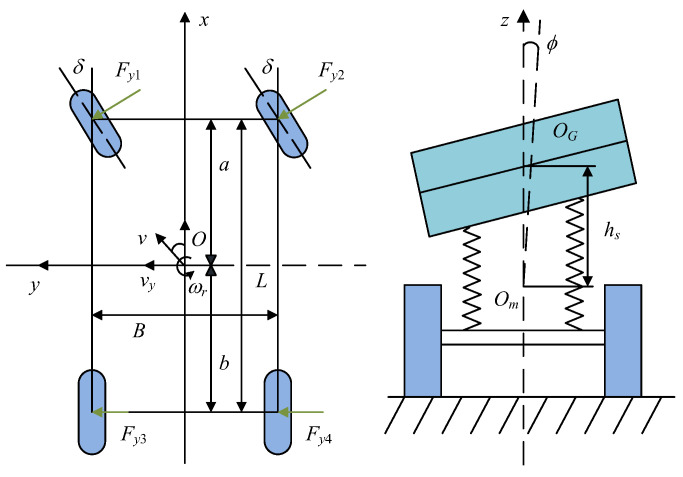
Vehicle dynamics model with three degrees of freedom.

Given that the path tracking accuracy and the body stability of IV are difficult to achieve under extreme working conditions, we need to consider the body posture of the vehicle. In this study, a three-degrees-of-freedom vehicle dynamic model [13] considering the body roll is established, as shown in Figure 2. This model ignores the longitudinal motion of the vehicle, the role of the suspension, and the change in unsprung mass.

The dynamic model of the vehicle can be described as

(1) $$\left\{ \begin{array}{l} m\left({\dot{v}}_{y}+v_{x}\omega_{r}\right)=\frac{\left(k_{1}+k_{2}\right)v_{y}}{v_{x}}+\frac{ak_{1}-bk_{2}}{v_{x}}\omega_{r}-\left(k_{1}E_{f}+k_{2}E_{r}\right)\phi -k_{1}\delta +m_{s}h_{s}\ddot{\phi }\\ I_{z}{\dot{\omega }}_{r}=\frac{\left(ak_{1}-bk_{2}\right)v_{y}}{v_{x}}+\frac{a^{2}k_{1}-b^{2}k_{2}}{v_{x}}\omega_{r}-\left(ak_{1}E_{f}-bk_{2}E_{r}\right)\phi -ak_{1}\delta \\ I_{x}\ddot{\phi }=m_{s}h_{s}\left({\dot{v}}_{y}+v_{x}\omega_{r}\right)+m_{s}h_{s}g\sin\phi -K\phi -C\dot{\phi } \end{array}\right.$$

where m and $m_{s}$ are the total mass and sprung mass of the vehicle, respectively; *a****_y_*** is lateral acceleration; *I****_x_*** and *I_Z_* are the moments of inertia about the axis X and axis Y, respectively; *K* and *C* are the equivalent roll stiffness and damping, respectively; $k_{1}$ and $k_{2}$ are the lateral deviation stiffness of the front and rear wheels, respectively; *E_f_* and *E_r_* are the equivalent roll steering coefficient of the front and rear axles, respectively; *a* and *b* are the distance between the centre of the mass of the vehicle and the front and rear axles, respectively; δ is the angle of the front wheel; $\omega_{r}$ is yaw rate; $h_{s}$ is the distance between the mass centre of spring and the roll axis; φ is the angle of roll; *v_x_* is the longitudinal speed of the vehicle; *v_y_* is the lateral speed of the vehicle.

#### 3.1.2. Preview Error Model

**Figure 3:**
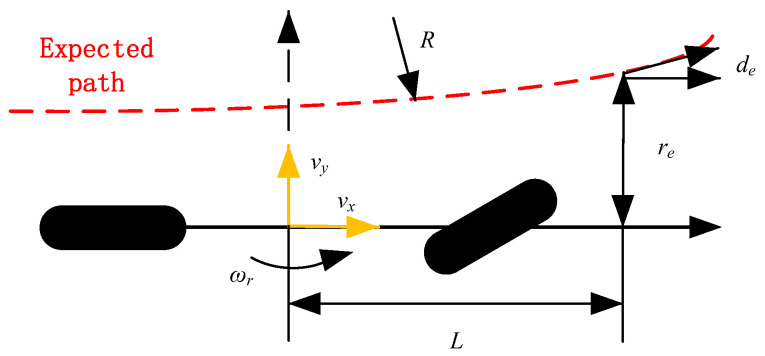
Schematic diagram of preview error model principle.

Amongst many tracking models, the execution logic of the preview error model [14] is the closest to the steering and tracking operation of the driver in the actual driving process. It also has good real-time performance and robustness. Thus, it is selected as the tracking model in the present study. Its basic principle diagram is as follows:

In Figure 3, *v_x_* represents the longitudinal speed of the vehicle; *v_y_* is the lateral speed of the vehicle; *ω_r_* signals the yaw rate of the car; *L* is the preview distance between the current position of the vehicle and the target position; *d_e_* is the lateral deviation; *r_e_* signals the direction deviation; *R* represents the radius of the desired path at this time, which is expressed in this article with the current position curvature *ρ*. The preview error model is as follows:

(2) $$\left\{ \begin{array}{l} {\dot{d}}_{e}=v_{y}+v_{x}r_{e}+L{\dot{r}}_{e}\\ {\dot{r}}_{e}=\omega_{r}-v_{x}\rho \end{array}\right.$$

After dimensionless processing, the two are combined into single-error data according to a certain weighting ratio. They are provided to the controller for path tracking, that is, the comprehensive error:

(3) $$E=\lambda {\bar{d}}_{e}+(1-\lambda ){\bar{r}}_{e}$$

where *λ* is the weight coefficient, ${\bar{d}}_{e}$ is the lateral deviation after normalization processing; ${\bar{r}}_{e}$ signals the direction deviation after normalization processing.

#### 3.1.3. Design of Fuzzy Sliding Mode Controller

The fuzzy sliding mode controller [15,16] consists of three parts: equivalent controller, switching controller, and fuzzy controller. The design process is as follows:

1. Design of Equivalent Control Unit

Constructing the equivalent control unit requires the selection of sliding mode functions, including traditional sliding mode surface, terminal sliding mode surface, and integral sliding mode surface. In this study, the traditional sliding mode surface design with a simple design and strong control applicability is selected. Defining switch function:

(4) $$s=\dot{E}+cE\;\left(c>0\right)$$

where *c* is the coefficient of sliding mode surface, E is the comprehensive error, and $\dot{E}$ is the derivative of the comprehensive error. 

When the sliding mode control reaches the ideal state, namely, the arrival condition of the sliding mode,

(5) $$\dot{s}=0$$

If solution $\delta_{eq}$ exists in Equation (5), the solution is the equivalent control of the system in the sliding mode region. This solution usually applies to the control system without external interference. In the actual system motion process, the process of the system motion point reaching the switching surface from any initial state is called approaching motion, that is, *s* is infinitely close to 0. Therefore, the following exponential reaching law is used to improve the dynamic quality of approaching motion in sliding mode motion:

(6) $$\dot{s}=-\eta sgn\left(s\right)-ks$$

In the formula, *η* and *k* both reach the law parameters, and *η* > 0 and *k* > 0. We can obtain the following by substituting Equation (6) into Equation (5) and then taking the derivative with respect to *s*:

(7) $$\dot{s}=\ddot{y}\frac{\lambda }{y_{max}}+\ddot{\theta }\frac{\left(1-\lambda \right)}{\theta_{max}}+\dot{y}\frac{c\lambda }{y_{max}}+\dot{\theta }\frac{c\left(1-\lambda \right)}{\theta_{max}}=-\eta sgn\left(s\right)-ks$$

In summary, equivalent control $\delta_{eq}$ can be obtained:

(8) $$\delta_{eq}=\frac{slaw-q_{1}v_{y}-q_{2}\omega_{r}-q_{3}\phi -q_{4}\dot{\phi }-q_{5}}{q_{6}}$$

where

$$\begin{array}{l} q_{1}=\frac{\lambda }{y_{max}}(\frac{I_{x}\left(k_{f}+k_{r}\right)}{v_{x}\left(mI_{x}-m_{s}{}^{2}h_{s}{}^{2}\right)}+\frac{-I_{x}\left(k_{f}E_{f}+k_{r}E_{r}\right)+m_{s}h_{s}\left(m_{s}h_{s}g-K\right)\left(ak_{f}-bk_{r}\right)}{I_{z}v_{x}\left(mI_{x}-m_{s}{}^{2}h_{s}{}^{2}\right)})+\frac{\left(1-\lambda \right)\left(ak_{f}-bk_{r}\right)}{\theta_{max}I_{z}v_{x}}+\frac{C\lambda }{y_{max}};\\ q_{2}=\frac{\lambda }{y_{max}}(\frac{\left(ak_{f}-bk_{r}\right)I_{x}-mv_{x}{}^{2}I_{x}+m_{s}{}^{2}h_{s}{}^{2}v_{x}{}^{2}}{v_{x}\left(mI_{x}-m_{s}{}^{2}h_{s}{}^{2}\right)}+v_{x}+\frac{a^{2}k_{f}-b^{2}k_{r}}{I_{z}v_{x}}C)+\frac{\left(1-\lambda \right)\left(a^{2}k_{f}-b^{2}k_{r}\right)}{\theta_{max}I_{z}v_{x}}+\frac{C\lambda L}{y_{max}}+\frac{C\left(1-\lambda \right)}{\theta_{max}};\\ q_{3}=\frac{\lambda }{y_{max}}(\frac{-I_{x}\left(k_{f}E_{f}+k_{r}E_{r}\right)+m_{s}h_{s}\left(m_{s}h_{s}g-K\right)}{mI_{x}-m_{s}{}^{2}h_{s}{}^{2}}+\frac{-\left(ak_{f}E_{f}-bk_{r}E_{r}\right)}{I_{z}}L)+\frac{-\left(1-\lambda \right)\left(ak_{f}E_{f}-bk_{r}E_{r}\right)}{\theta_{max}I_{z}};\\ q_{4}=\frac{\lambda }{y_{max}}\frac{-Cm_{s}h_{s}}{mI_{x}-m_{s}{}^{2}h_{s}{}^{2}};q_{5}=\frac{\lambda v_{x}{}^{2}\rho }{y_{max}}+\frac{c\lambda }{y_{max}}\left(v_{x}\theta -Lv_{x}\rho \right)-\frac{c\left(1-\lambda \right)v_{x}\rho }{\theta_{max}};q_{6}=\frac{\lambda }{y_{max}}\left(\frac{-k_{f}}{m}+\frac{-ak_{f}}{I_{z}}C\right)+\frac{1-\lambda }{\theta_{max}}\frac{-ak_{f}}{I_{z}} \end{array}$$

2. Design of Switching Control Unit

After the system state is kept on the sliding mode surface by equivalent control, switching control is required to ensure that the system state slides up and down on the switching surface. It can also improve the robustness of the system and reduce the uncertainty in the control process. In this case, the control law of equivalent control plus switching control is as follows:

(9) $$u=u_{eq}+u_{vss}$$

The design of the switching controller $u_{vss}$ is as follows:

(10) $$u_{vss}=\frac{1}{g(x,t)}\eta sgn(s)$$

We can obtain the following by substituting the equation $q_{6}$ into the above equation:

(11) $$\delta_{sw}=\frac{1}{\frac{\lambda }{y_{max}}\left(\frac{-k_{f}}{m}+\frac{-ak_{f}}{I_{z}}C\right)+\frac{1-\lambda }{\theta_{max}}\frac{-ak_{f}}{I_{z}}}\eta sgn(s)$$

The final sliding mode controller is

(12) $$\delta =\delta_{eq}+\delta_{sw}$$

3. Design of Fuzzy Control Unit

In the fuzzy sliding mode controller, the input of the fuzzy controller is the sliding mode surface S(t). In the control process, the fuzzy control [17,18,19] can adjust the equivalent control part and switching control part in the sliding mode controller according to the state of the sliding mode surface. In particular, the switching control must be added through the fuzzy control when the system state is far from the sliding mode surface. The original equivalent part can be maintained when the system is close to the sliding mode surface. Based on this scenario, the control rules are as follows:

$$\begin{array}{l} \;\mathrm{If}\;S(t)\;\mathrm{is}\;\mathrm{ZO}\;\mathrm{then}\;\delta \;\mathrm{is}\;\delta_{eq}\\ \mathrm{If}\;S(t)\;\mathrm{is}\;\mathrm{NZ}\;\mathrm{then}\;\delta \;\mathrm{is}\;\delta_{eq}+\delta_{sw} \end{array}$$

where S(t) represents the real-time state of the sliding mode surface, and ZO and NZ represent ‘zero’ and ‘nonzero’, respectively. The membership functions confirmed by the fuzzy statistical method are TRIMF, SMF, and ZMF.

According to the fuzzy rules, the fuzzy controller needs only the equivalent control $\delta_{eq}$ when the sliding mode holds. The fuzzy controller consists of equivalent control $\delta_{eq}$ and switching control $\delta_{sw}$ when the system state does not reach the sliding mode surface.

After defuzzification, the fuzzy controller is

(13) $$\delta =\frac{\mu_{ZO}(s)\delta_{eq}+\mu_{NZ}(s)\left(\delta_{eq}+\delta_{SW}\right)}{\mu_{ZO}(s)+\mu_{NZ}(s)}=\delta_{eq}+\mu_{NZ}(s)\delta_{{}_{SW}}$$

(14) $$\mu_{ZO}(s)+\mu_{NZ}(s)=1$$

Therefore, the control law is a normal equivalent sliding mode control when the membership function $\mu_{NZ}(s)$ equals 1. However, when it is not equal to 1, the influence of chattering can be weakened by the change in the membership function $\mu_{NZ}(s)$.

### 3.2. Design of Curvature Optimisation System 

The realisation method of the curvature optimisation system (shown in Figure 1) analyses the body stability under different working conditions on the basis of the completed path tracking control system. This method also determines the stability constraint boundary conditions in the optimisation process with the help of the vehicle yaw rate, lateral acceleration, and roll angle. Then, the optimisation algorithm finds the actual driving path $\rho_{2}{}^{*}$ closest to the original desired path within the constraint range according to the known expected path curvature $\rho_{1}$ and $d_{e1}$ under this working condition. Moreover, the algorithm inputs the corresponding optimised expected path $\rho_{2}$ into the path tracking controller. The controller uses the path as the tracking path to retrack the control. The tracking error at this time is reduced from $d_{e1}$ to $\left|d_{e}\right|$ to improve tracking accuracy. The stability constraint also ensures that the body stability changes within a reasonable range to achieve a comprehensive improvement of the tracking effect.

#### 3.2.1. Determination of Stability Boundary Conditions

In this study, the stability boundary conditions are determined from the three angles of yaw rate, lateral acceleration, and roll angle [20,21]. In studying the vehicle stability region boundary, the range of yaw rate in the stability boundary condition is [−1.05, 1.05] [22]. Lateral acceleration is an important index in the stability evaluation system [23]. In particular, it can be used as a separate rollover threshold to analyse the rollover problem under vehicle instability. The limit value of lateral acceleration in the stability boundary condition is set as 0.4 g to reasonably ensure the smooth running of the vehicle [24]. The roll angle is an important factor for judging the vehicle’s body stability. The most appropriate roll angle is 3° when the lateral acceleration is 0.4 g. Moreover, the maximum roll angle should not exceed 5°. In this study, the stability limit value considered in the curvature optimisation process is 5° [25].

**Figure 4:**
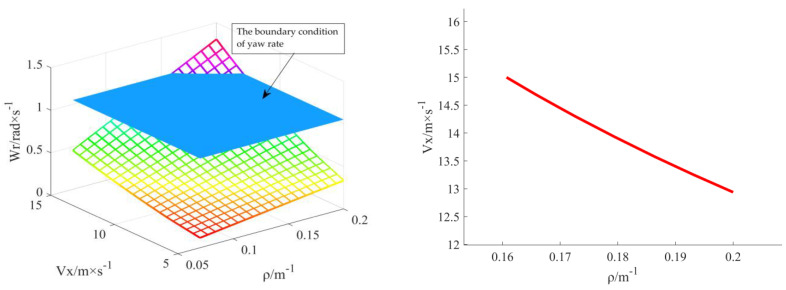
Curvature boundary map under yaw rate constraint.

Figure 4 shows that the yaw rate change surface intersects the stability boundary surface within the range of high speed and large curvature. The yaw rate may exceed the set of 1.05 rad/s when the vehicle speed exceeds 13.08 m/s. Thus, body stability is affected. The critical value of curvature is 0.162 m^−1^. Therefore, the curvature optimisation algorithm limited by yaw rate should set the boundary value of curvature only as 0.162 m^−1^ in the speed range [5,15] set in this study.

**Figure 5:**
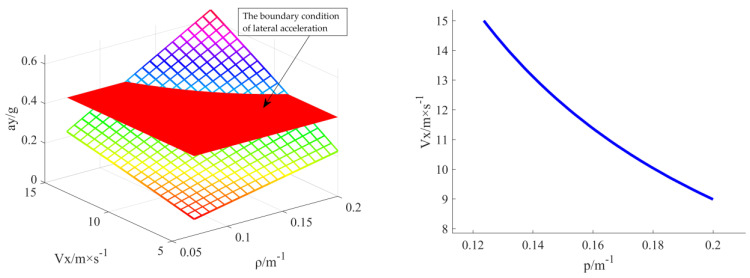
Curvature boundary map under lateral acceleration constraint.

Figure 5 shows that the boundary values of the intersection line between the lateral acceleration response surface and the stability boundary surface are 0.124 m^−1^ and 9.262 m/s. This finding indicates that if the speed is lower than 9.262 m/s or the curvature is less than 0.124 m^−1^, the lateral acceleration of the vehicle cannot be greater than the set of 0.4 g, no matter how the curvature is adjusted. The optimal boundary value under the lateral acceleration limit should be 0.124 m^−1^ to ensure the stable driving of the vehicle.

**Figure 6:**
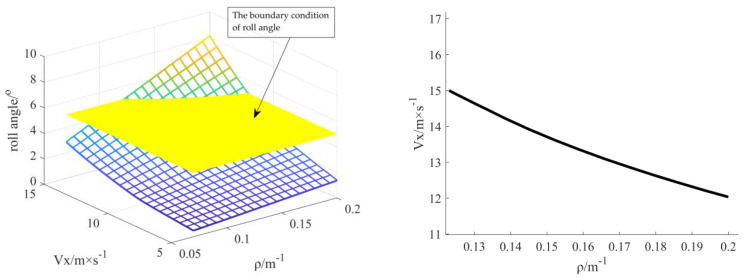
Curvature boundary map under roll angle constraint.

Figure 6 shows that the roll angle of the vehicle is stable at low speed and does not exceed the critical value of 5°. Once the vehicle speed reaches the boundary value of 12.15 m/s, the curvature optimisation is suddenly limited by the roll angle. The curvature must be lower than 0.127 m^−1^ when the vehicle speed reaches the limit of 15 m/s to ensure that the vehicle roll angle is within the acceptable range. Therefore, the curvature optimisation boundary condition of the proposed model is 0.127 m^−1^ to ensure the stability of the vehicle body under the limitation of roll angle.

#### 3.2.2. Algorithm Optimisation

The curvature optimisation method based on a genetic algorithm is adopted in this study. The basic idea of the genetic algorithm [26,27] is derived from the viewpoint of Darwinian evolution, that is, the survival of the fittest. Crossover and mutation make the gene close to the optimal solution in the inheritance process through selection. Then, the optimisation process is realised. The genetic algorithm has the advantages of strong robustness, minimal conditions required for optimisation, and easy implementation. Thus, it is widely used in various fields [28,29]. The optimisation steps of the genetic algorithm in this study are as follows:

1. Encoding and Decoding

The genetic algorithm cannot directly deal with the parameters to be optimised in the actual control model. Therefore, coding must be used to transform data into strings composed of specific symbols in a certain order, like how chromosomes are synthesised from genomes. The coding method affects the operation of subsequent operators. The effect largely determines the final optimisation efficiency. Common coding methods include the binary coding method, the Gray coding method, the floating point coding method, and the symbolic coding method; the commonly used binary coding is selected here [30]. Binary encoding transforms the optimisation parameters into 0, 1 for representation. The optimisation parameter in the present study is the curvature of the desired path *ρ*. The length l of the binary string is calculated by the following formula:

(15) $$l=\log_{2}\left(\frac{b-a}{eps}\right)+1$$

where *a* (0.07) and *b* (0.2) are the ranges of the optimisation parameters; *eps* is the required accuracy of optimisation, and 0.01 is chosen here. After being obtained, the final optimisation parameters must be decoded and output. The formula is as follows:

(16) $$x=a+(b-a)\frac{X}{2^{l}-1}$$

In the above formula, *X* is the binary expression of optimisation parameters, and x is the final decimal expression.

2. Initializing the Population and Fitness Function

In general, the initial population of genetic algorithms is randomly generated. Only a certain number of individuals are randomly generated within the range of the optimal solution for iteration.

The fitness function of the genetic algorithm determines that the probability of an individual can be inherited. The larger the value of the fitness function, the more suitable the individual is to the elimination process, and the greater the probability of producing subindividuals is. Therefore, the fitness function is a kind of evaluation index of individual merits. The design of the fitness function also affects the optimisation speed and efficiency of the genetic algorithm.

The optimisation process in this study finds the target path closest to the desired path for tracking, which is the problem of finding the minimum value. Thus, it needs to be scaled:

(17) $$f(x)=\frac{1}{F}$$

where f(x) is the fitness function, and the design process of evaluation index F is as follows:

(18) $$F=\left|\frac{1}{\rho_{2}}-d_{e2}-\frac{1}{\rho_{1}}\right|$$

where *ρ*_2_ and *ρ*_1_ are the curvature of the optimised path and the expected tracking path, respectively, and $d_{e2}$ is the transverse deviation of the corresponding path. In the fitness function, the optimised curvature should also meet the boundary condition requirements of the body stability:

(19) $$\left\{ \begin{array}{l} \omega_{rf}\leq \omega_{r\max}\\ a_{yf}\leq a_{y\max}\\ \phi_{f}\leq \phi_{\max} \end{array}\right.$$

3. Selecting Operator

In the genetic algorithm, the superior individual must be selected from the parent generation for inheritance. The judgment of an individual’s merits and demerits is determined by its fitness. Thus, the selection operation is equivalent to the survival of the fittest in the evolutionary process, which affects the final convergence of the algorithm. According to the roulette selection method for operator selection, we can obtain the selection probability $p(x_{i})$ of individual $x_{i}$ as follows:

(20) $$p(x_{i})=\frac{f(x_{i})}{\sum_{j=1}^{N}f(x_{j})}$$

The calculation formula of chromosome $q(x_{i})$ is as follows:

(21) $$q(x_{i})=\sum_{j=1}^{i}p(x_{j})$$

4. Crossover Operator

The crossover operation process is used to generate new individuals. This process is also a key step different from other evolutionary algorithms. It is realised by the random exchange of gene information between individuals. Some new dominant individuals are generated because the original dominant individuals are retained, thereby improving the search speed of the whole population of genetic algorithms.

5. Mutation Operator

The mutation operator in the genetic algorithm can avoid the elimination of some dominant gene codes in advance. It can prevent prematureness, ensure population diversity, and make the genetic algorithm obtain local random search ability to accelerate the search speed.

In this study, the basic bit mutation operator is used to mutate the random one-bit or multibit gene code of an individual according to the set mutation probability. In particular, the binary coding in this study converts the 0 and 1 of the mutation bit.

**Figure 7:**
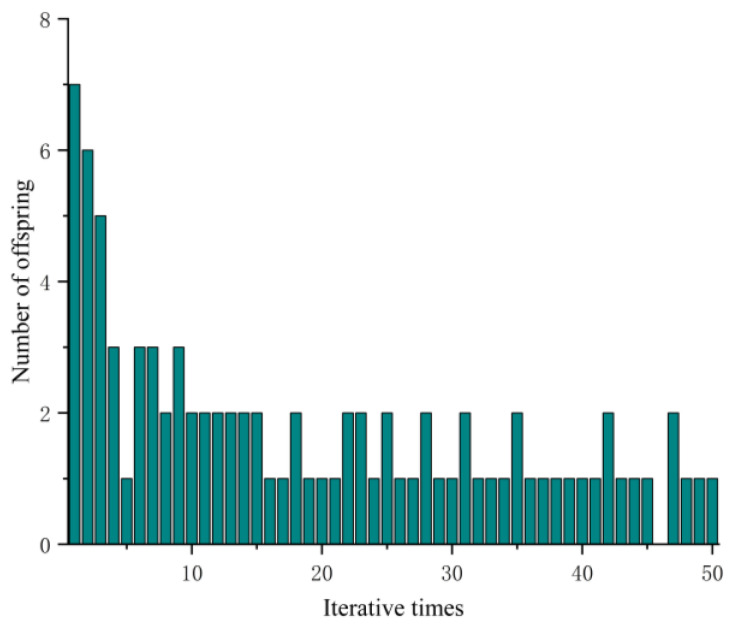
Schematic diagram of the genetic algorithm optimisation process.

After the basic steps of the genetic algorithm, the optimisation is determined, and the working condition of *ρ*_1_ = 0.1 m^−1^ and *v_x_* = 10 m/s is selected for curvature optimisation simulation. The schematic diagram of the optimisation process is as follows:

Figure 7 shows the number of offspring generated under different genetic generations. The iteration progress makes the number of offspring gradually stabilise and tend to 1, indicating the end of the optimisation process. 

## 4. Analysis of Simulation Result

The focus of this paper is that a path tracking control method based on curvature optimisation is designed to solve the deterioration of vehicle stability even when the path tracking accuracy of the IV is improved. Therefore, the longitudinal speed of the vehicle studied in this paper is the constant speed condition. Lateral deviation, directional deviation, yaw rate, lateral acceleration, and roll angle are selected as evaluation indexes in the present study to verify the effectiveness of the proposed path following the control method based on curvature optimisation. Firstly, the tracking effect of the controller before and after optimisation is verified by different working conditions. Secondly, the optimised tracking effects under different curvatures, different vehicle speeds, and mixed conditions are selected for comparative analysis.

Some parameters of the vehicle in the simulation are shown in Table 1.

### 4.1. Comparison before and after Optimisation

**Figure 8:**
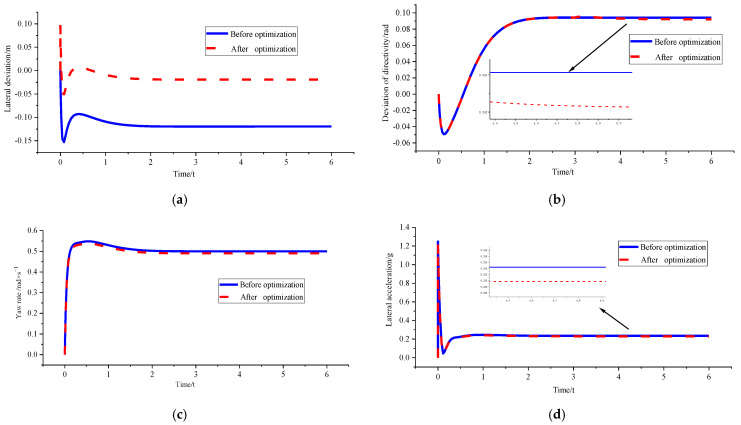

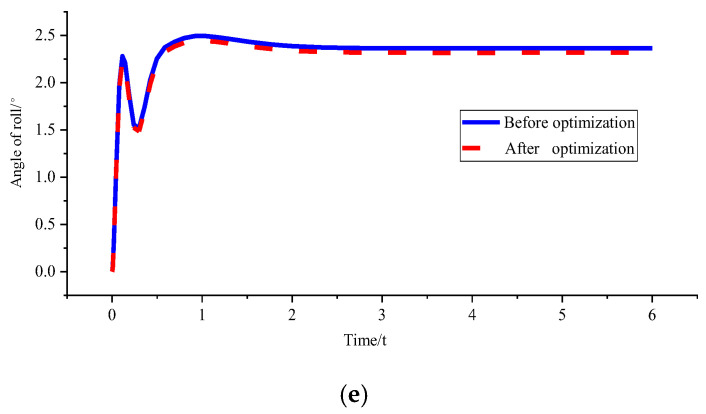
Comparison of path tracking effects before and after optimisation under *v_x_* = 10 m/ s and *ρ* = 0.15 m^−1^. (**a**) Comparison chart of lateral deviations. (**b**) Comparison chart of direction deviation. (**c**) Comparison chart of yaw rate. (**d**) Comparison chart of lateral acceleration. (**e**) Comparison chart of roll angles.

**Figure 9:**
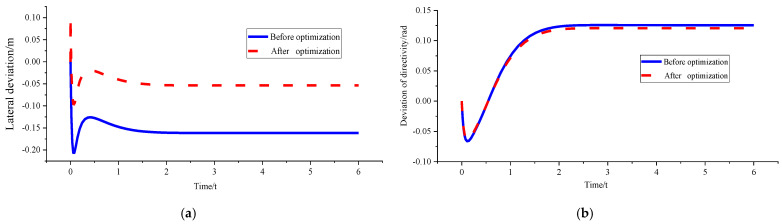

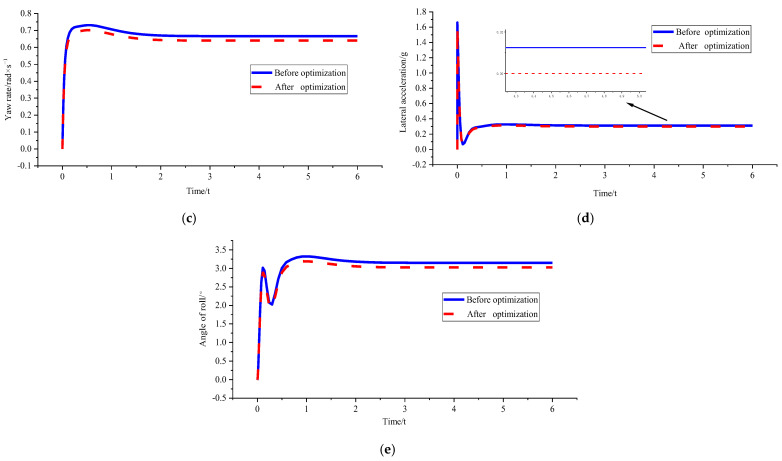
Comparison of path tracking effect before and after optimisation under *v_x_* = 10 m/s and *ρ* = 0.2 m^−1^. (**a**) Comparison chart of lateral deviations. (**b**) Comparison chart of direction deviation. (**c**) Comparison chart of yaw rate. (**d**) Comparison chart of lateral acceleration. (**e**) Comparison chart of roll angles.

**Figure 10:**
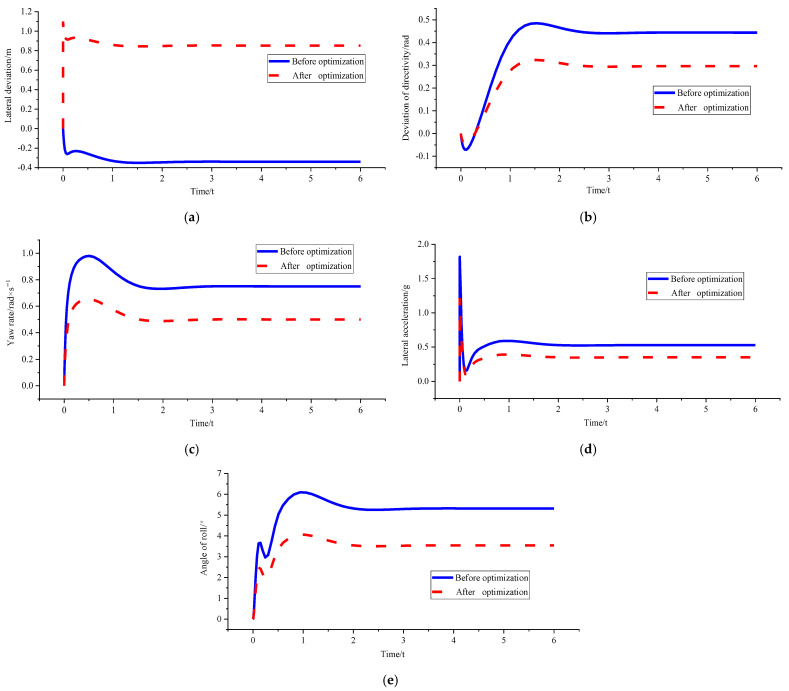
Comparison of path tracking effects before and after optimisation under *v_x_* = 15 m/s and *ρ* = 0.15 m^−1^. (**a**) Comparison chart of lateral deviations. (**b**) Comparison chart of direction deviation. (**c**) Comparison chart of yaw rate. (**d**) Comparison chart of lateral acceleration. (**e**) Comparison chart of roll angles.

In order to verify the superiority of the control algorithm under different speed and different curvature, *v_x_* = 10 m/s and *ρ* = 0.15 m^−1^, *v_x_* = 10 m/s and *ρ* = 0.2 m^−1^, and *v_x_* = 15 m/s and *ρ* = 0.15 m^−1^ were used for simulation verification. Figure 8, Figure 9 and Figure 10 shows the simulation results. Table 2, Table 3 and Table 4 shows the comparison results of lateral deviations, direction deviation, yaw rate, lateral acceleration, and roll angles under different vehicle speed and different curvature.

(1) *v_x_* = 10 m/s and *ρ* = 0.15 m^−1^

Figure 8 and Table 2 show that the lateral deviation is reduced from 0.11955 m to 0.01901 m, the optimisation amplitude is 84.10%, and the optimisation result of the stability parameter is only increased by 2.00%. The reason is that the vehicle is in a low-speed and low-curvature condition, and the optimised curvature is not much different from the original curvature. Therefore, the changes in the stability of the body can be almost ignored without stability consideration.

**Table 2:** Parameter peaks before and after path tracking optimisation under *v_x_* = 10 m/s and *ρ* = 0.15 m^−1^.

	*d_e_* (m)	*r_e_* (rad)	*ω_r_* (rad/s)	*a_y_* (g)	*ϕ* (°)
Before optimisation	−0.11955	0.094130	0.548289	0.245355	2.492409
After optimisation	0.01901	0.092247	0.537309	0.240448	2.442508
Percentage of optimisation	84.10	2.00	2.00	2.00	2.00

(2) *v_x_* = 10 m/s and *ρ* = 0.2 m^−1^

Figure 9 and Table 3 show that the optimised curvature obtained by the genetic algorithm is 0.192 m^−1^, and the corresponding lateral deviation optimisation percentage is 66.80%. However, the optimisation degree of stability parameters is not large at around 4%. However, the optimisation effect under the same vehicle speed and large curvature is more obvious than that under the same vehicle speed and small curvature.

**Table 3:** Parameter peaks before and after path tracking optimisation under *v_x_* = 10 m/s and *ρ* = 0.2 m^−1^.

	*d_e_* (m)	*r_e_* (rad)	*ω_r_* (rad/s)	*a_y_* (g)	*ϕ* (°)
Before optimisation	−0.16143	0.125506	0.73114	0.327183	3.017048
After optimisation	−0.0536	0.120486	0.701989	0.314127	2.905264
Percentage of optimisation	66.80	3.40	3.99	3.99	3.71

(3) *v_x_* = 15 m/s and *ρ* = 0.15 m^−1^

Figure 10 and Table 4 show that the genetic algorithm directly sets the optimised curvature as the corresponding critical curvature of 0.124 m^−1^ under *v_x_* = 15 m/s because of the boundary condition limitation triggered by the peak value of lateral acceleration and roll angle. At this time, the yaw rate, lateral acceleration, and roll angle have all been optimised to some extent. The final value is stable within the boundary range, which ensures the stable driving of the vehicle in the path tracking process. However, part of the lateral tracking performance is sacrificed because the tracking path has deviated from the initially set desired path. Moreover, the final lateral deviation is 0.78213 m, which is still within the acceptable range.

**Table 4:** Parameter peaks before and after path tracking optimisation under *v_x_* = 15 m/s and *ρ* = 0.15 m^−1^.

	*d_e_* (m)	*r_e_* (rad)	*ω_r_* (rad/s)	*a_y_* (g)	*ϕ* (°)
Before optimisation	−0.33986	0.443912	0.980035	0.589204	6.095175
After optimisation	0.78213	0.295941	0.653149	0.392813	4.066013
Percentage of optimisation	−130.13	22.28	33.35	33.33	33.29

In summary, the curvature optimisation controller designed based on the fuzzy sliding mode path tracking controller in this study can improve tracking accuracy, ensure the stable driving of vehicles, and achieve the desired tracking effect. This controller also has high feasibility.

### 4.2. Comparative Analysis of Tracking Effect after Optimisation

After determining the feasibility of the curvature optimization method, in order to further analyse the optimization effect, different curvature conditions, different speed conditions, and mixed conditions were selected for comparative analysis of the optimized tracking effect. Curvature was selected as 0.15 m^−1^ and 0.2 m^−1^, and speed was selected as 10 m/s and 15 m/s. The results are as follows:

(1) *v_x_* = 10 m/s and *ρ* = 0.15 m^−1^; *v_x_* = 10 m/s and *ρ* = 0.2 m^−1^

**Figure 11:**
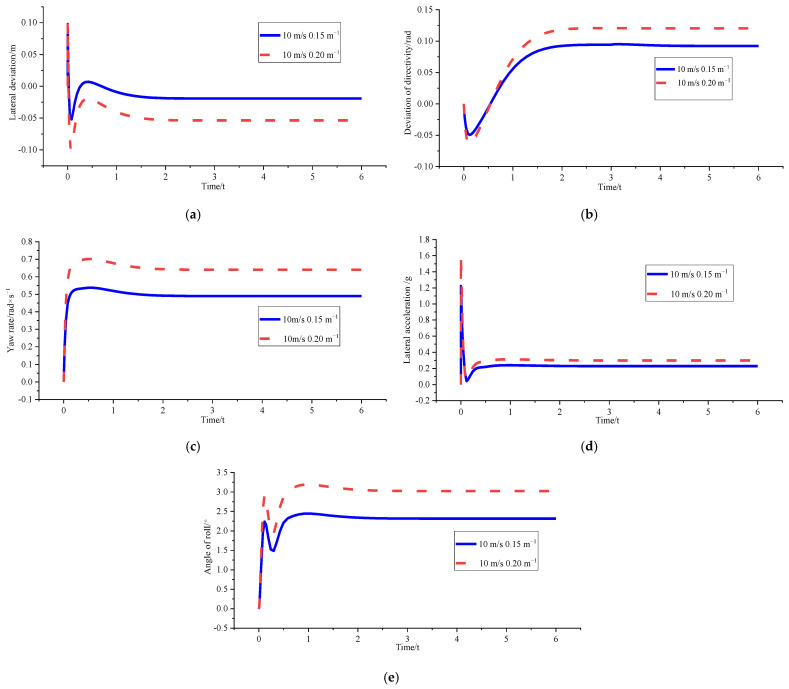
Comparison of optimised effects with different curvature. (**a**) Comparison chart of lateral deviations. (**b**) Comparison chart of direction deviation. (**c**) Comparison chart of yaw rate. (**d**) Comparison chart of lateral acceleration. (**e**) Comparison chart of roll angles.

Figure 11 and Table 5 show that fixed speed improved curvature results in increased optimised tracking error. This trend is the same as that of the fuzzy sliding mode path-following controller without optimisation. It also illustrates the rationality of the optimisation method of curvature. The comparison of the stability parameters of the two kinds of curvatures indicates that the optimised lateral accelerations of the two are close to each other, and the variation trend is reasonable. Moreover, they are within the set stability boundary range without triggering stability constraints.

(2) *v_x_* = 10 m/s and *ρ* = 0.15 m^−1^; *v_x_* = 15 m/s and *ρ* = 0.15 m^−1^

**Figure 12:**
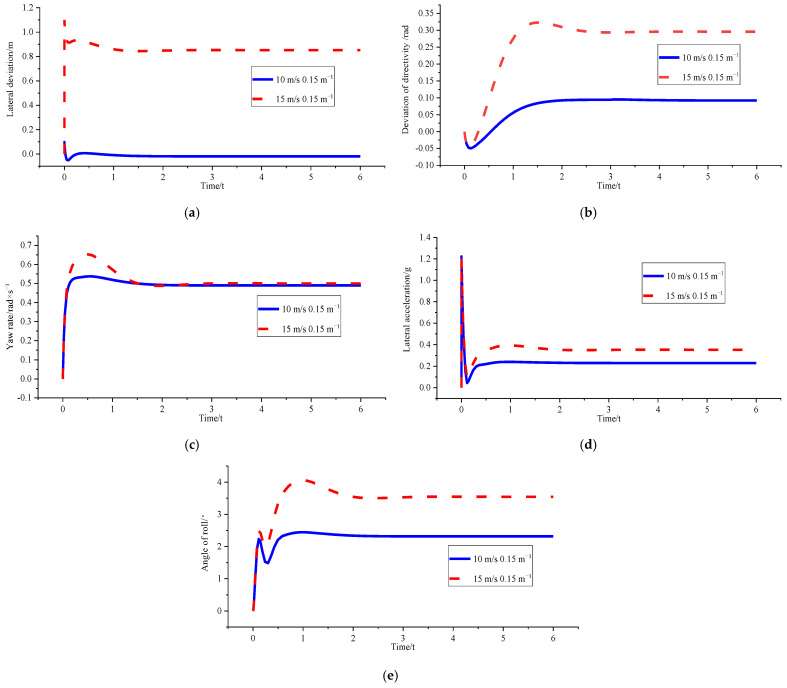
Comparison of optimised effects of different vehicle speeds. (**a**) Comparison chart of lateral deviations. (**b**) Comparison chart of direction deviation. (**c**) Comparison chart of yaw rate. (**d**) Comparison chart of lateral acceleration. (**e**) Comparison chart of roll angles.

Figure 12 and Table 6 show that after curvature optimisation, the lateral deviation at 10 m/s is small enough to be ignored. This finding indicates that curvature optimisation can achieve high-precision tracking control, whereas the stability constraint in the optimisation algorithm is triggered at 15 m/s. Moreover, adjusting the stability of the body is the primary objective. According to the results, stability regulation is indeed achieved.

(3) *v_x_* = 10 m/s and *ρ* = 0.15 m^−1^; *v_x_* = 15 m/s and *ρ* = 0.15 m^−1^ ; *v_x_* = 10 m/s and *ρ* = 0.2 m^−1^

**Figure 13:**
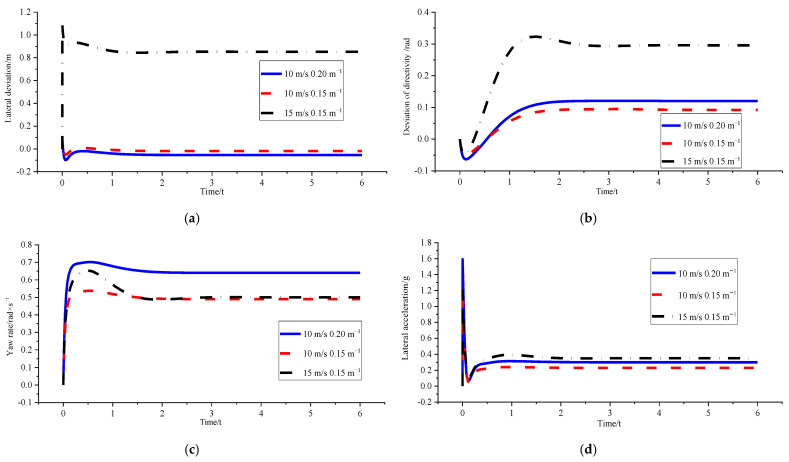

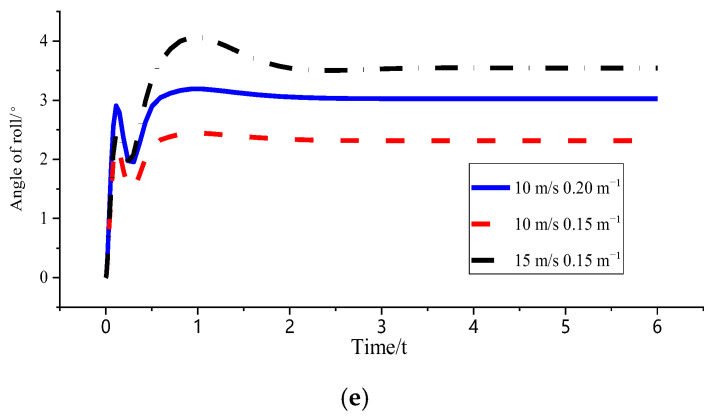
Comparison of optimised effects after hybrid working conditions. (**a**) Comparison chart of lateral deviations. (**b**) Comparison chart of direction deviation. (**c**) Comparison chart of yaw rate. (**d**) Comparison chart of lateral acceleration. (**e**) Comparison chart of roll angles.

Figure 13 and Table 7 show that the tracking accuracy comparison of the optimised mixed condition indicates that the optimisation effect is the best under the 10 m/s and 0.15 m^−1^ condition_._ The change in tracking deviation when the vehicle speed increases after optimisation is greater than the effect caused by the increase in curvature. The comparison of the optimised vehicle stability under mixed working conditions shows that the stability boundary conditions triggered by the high-speed working condition of 15 m/s make the yaw velocity lower than 10 m/s. The values of other stability evaluation parameters are also close to those of the 10 m/s working condition, indicating the feasibility of stability constraint design in the curvature optimisation system in the simulation. It can realise the comprehensive improvement of tracking accuracy and body stability.

In summary, the simulation comparison results after optimisation show that the values of the precision parameters and stability parameters under any working condition are within a reasonable range. This finding proves the effectiveness of the optimised controller. The influence of the speed and curvature changes on the tracking effect is consistent with that of the fuzzy sliding mode controller. Under different working conditions, the optimisation results of low speed and low curvature are good, indicating that the curvature optimisation does not affect the coupling relationship between the tracking effect of the original path tracking controller and the speed and curvature.

## 5. HIL Test Verification

**Figure 14:**
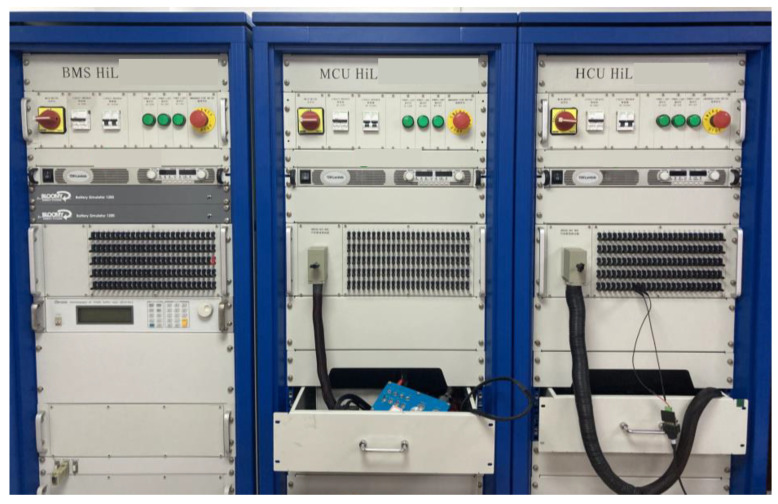
Structure diagram of the HIL system development test bench.

The theory of the HIL simulation test uses various boards to simulate the input and output signals of the IV path tracking system. Moreover, a simulation analysis is conducted through the effective connection between the controlled object model and the actual controller. The hardware in the loop test is safer than the real vehicle test, and the implementation of the control algorithm is relatively easy. This technology is not limited by external conditions, such as test conditions, test roads, and nearby environments. It can simulate the nonlinear factors and the dynamic and static real-time characteristics of IVs through the upper computer. The HIL test platform used in this study is shown in Figure 14.

The complexity of the real road conditions and the interference of the external environment are considered to verify further the effectiveness of the proposed path tracking control method based on curvature optimisation. The interference and influence of the system controller time delay on the IV path tracking system are impossible to verify accurately. Moreover, realising the control algorithm in the real vehicle test is difficult. This section is based on the HIL test platform. Based on the HIL test platform, the simulation in the previous section is tested under single and mixed conditions. The verification results are consistent with the theory.

The purpose of the single condition HIL test is to verify the optimization effect of the curvature optimisation controller on the real controller under different working conditions. The speed is selected as 15 m/s, and the curvature is selected as 0.15 m^−1^. The specific optimization effect is as follows:

(1) *v_x_* = 15 m/s and *ρ* = 0.15 m^−1^

**Figure 15:**
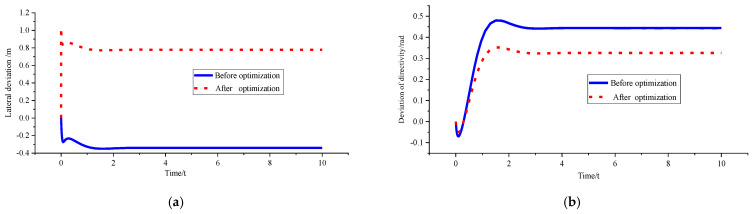

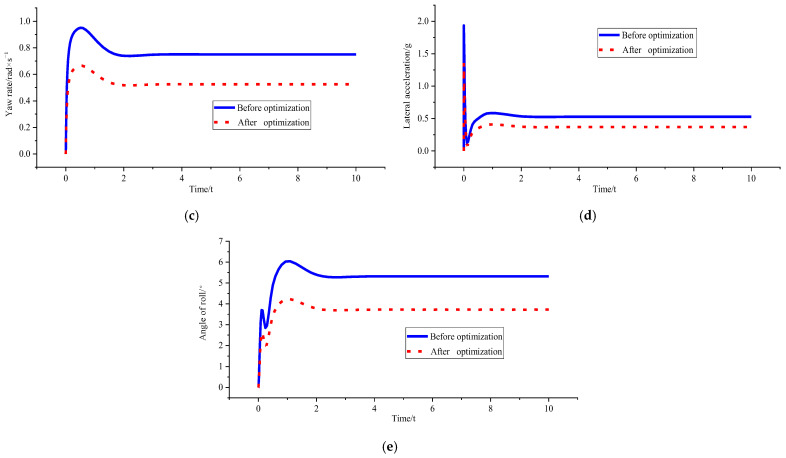
Comparison of hardware effects before and after loop test optimisation under *v_x_* = 15 m/s and *ρ* =0.15 m^−1^. (**a**) Comparison chart of lateral deviations. (**b**) Comparison chart of direction deviation. (**c**) Comparison chart of yaw rate. (**d**) Comparison chart of lateral acceleration. (**e**) Comparison chart of roll angles.

Figure 15 and Table 8 show that the stability boundary constraint in the physical controller is triggered, and the yaw rate, lateral acceleration, and roll angle are greatly optimised. We can find that the maximum value of lateral acceleration is 0.4091 g in the table. This value is larger than the 0.4 g boundary condition set in the algorithm. This scenario may be caused by signal conversion and transmission problems. The actual operation of the electronic components still exhibits some deviations within the acceptable range. Compared with the simulation optimisation effect, the optimisation percentage is also very close, indicating that the control effect of the optimisation algorithm in the physical controller under extreme working conditions reaches the expected goal.

The mixed conditions HIL test can effectively explore the influence of different curvature and speed change on the tracking effect of physical controller. For the convenience of comparison, the three conditions of *v_x_* = 10 m/s and *ρ* = 0.2 m^−1^; *v_x_* = 10 m/s and *ρ* = 0.15 m^−1^; *v_x_* = 15 m/s and *ρ* = 0.15 m^−1^ were selected for comparative analysis after optimization effect, the results are as follows:

(2) *v_x_* = 10 m/s and *ρ* = 0.2 m^−1^; *v_x_* = 10 m/s and *ρ* = 0.15 m^−1^; *v_x_* = 15 m/s and *ρ* = 0.15 m^−1^

**Figure 16:**
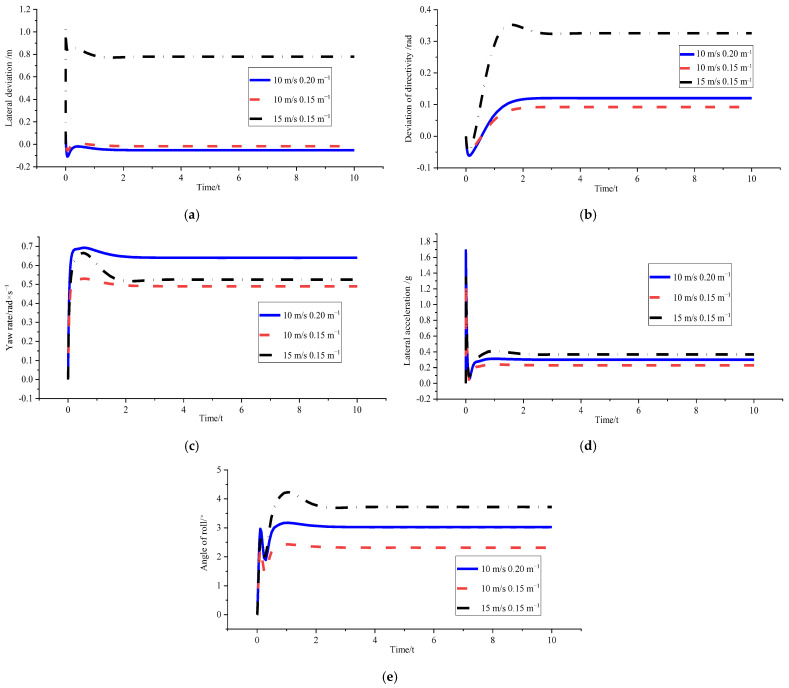
Comparison of hardware effects after loop test optimisation under multiple working conditions. (**a**) Comparison chart of lateral deviations. (**b**) Comparison chart of direction deviation. (**c**) Comparison chart of yaw rate. (**d**) Comparison chart of lateral acceleration. (**e**) Comparison chart of roll angles.

Figure 16 and Table 9 show that amongst the three working conditions, the optimisation effect is the best under the *v_x_* = 10 m/s and *ρ* = 0.15 m^−1^ condition. In particular, the fixed speed reduces the curvature, the tracking accuracy is improved, and the body stability performs well in the hardware in the loop test. The stability constraint is triggered when the speed reaches 15 m/s. The influence of vehicle speed variation under fixed curvature on the optimisation results cannot be compared. However, the figure shows that the yaw velocity is optimised well under stability regulation.

In summary, the effectiveness and feasibility of the whole designed control system in the physical controller can be proved by comparing the simulation test of the path tracking control system under different working conditions and the comparative analysis of the control effect of the optimisation algorithm under the loop test of the hardware.

## 6. Conclusions

This study designs a path tracking control system based on curvature optimisation. The coupling relationship between path tracking accuracy and vehicle body stability is considered starting from the actual situation of the path tracking process of the IV. Based on the path tracking control system of the fuzzy sliding film controller, the curvature optimisation algorithm is added to realise the dual optimisation of the IV path tracking. In the range of stability boundary threshold, the desired path with a low error can be obtained by curvature optimisation, and the tracking accuracy can be improved. The stability control mechanism is triggered when the state parameters of the vehicle exceed the stability threshold range, effectively adjusting the attitude of the vehicle. The simulation results show that under the *v_x_* = 10 m/s and *ρ* = 0.15 m^−1^ condition, the optimisation amplitude of lateral deviation is up to 84.10%, and the stability is improved by approximately 2%. Under the *v_x_* = 10 m/s and *ρ* = 0.2 m^−1^ condition, the optimisation amplitude of lateral deviation is up to 66.80%, and the stability is improved by approximately 4%. Under the *v_x_* = 15 m/s and *ρ* = 0.15 m^−1^ condition, the boundary conditions of body stability are triggered, and the body stability is improved by 20–30%. However, the tracking accuracy is lost, and the lateral deviation is 0.7821 m, which is within the acceptable range. The simulation comparison results after optimisation show that the values of the precision parameters and stability parameters under any working condition are within a reasonable range. This finding proves the effectiveness of the optimised controller. The influence of the speed and curvature changes on the tracking effect is consistent with that of the fuzzy sliding mode controller. Under different working conditions, the optimisation results of low speed and low curvature are good, indicating that the curvature optimisation does not affect the coupling relationship between the tracking effect of the original path tracking controller and the speed and curvature. The HIL test results further verify that the designed curvature optimisation method can effectively improve the tracking accuracy in the tracking process and ensure body stability to achieve the comprehensive improvement of the tracking effect.

## Figures and Tables

**Table 1:** Vehicle parameters.

Arguments	Quantitative Value
Vehicle curb weight m/kg	1495
Sprung mass m/kg	1335.6
The distance between the centre of mass and the anterior and posterior axes (a, b)/m	1.071, 1.529
The distance between the mass centre of spring and the roll axis $h_{s}$/m	0.488
The moment of inertia of the vehicle about the *x*-axis *I_x_*/(kg·m^2^)	730.95
The moment of inertia of the vehicle about the *z*-axis *I_z_*/(kg·m^2^)	3053.6
Equivalent lateral stiffness of the front and rear wheels *(k*_1_, *k*_2_)/(N/rad)	−23,147, −38,138
Equivalent roll damping C/(N·s/m)	6860
Equivalent roll stiffness *K*/(N·rad^−1^)	133,280
The equivalent roll steering coefficient of the front and rear axles *E_f_*, *E_r_*	−0.114, 0

**Table 5:** Comparative analysis of tracking effect of different curvature after optimization.

	*d_e_* (m)	*r_e_* (rad)	*ω_r_* (rad/s)	*a_y_* (g)	*ϕ* (°)
*v_x_* = 10 m/s *ρ* = 0.15 m^−1^	−0.01914	0.09228	0.48996	0.22967	2.31576
*v_x_* = 10 m/s *ρ* = 0.2 m^−1^	−0.05362	0.12050	0.63999	0.29999	3.02455

**Table 6:** Comparative analysis of tracking effect of different speed after optimization.

	*d_e_* (m)	*r_e_* (rad)	*ω_r_* (rad/s)	*a_y_* (g)	*ϕ* (°)
*v_x_* = 10 m/s *ρ* = 0.15 m^−1^	−0.01917	0.09261	0.48998	0.22968	2.31578
*v_x_* = 15 m/s *ρ* = 0.15 m^−1^	0.85234	0.29604	0.49991	0.35162	3.54543

**Table 7:** Comparative analysis of tracking effect of different speed and different curvature after optimization.

	*d_e_* (m)	*r_e_* (rad)	*ω_r_* (rad/s)	*a_y_* (g)	*ϕ* (°)
*v_x_* = 10 m/s *ρ* = 0.15 m^−1^	−0.01914	0.09228	0.48996	0.22967	2.31576
*v_x_* = 15 m/s *ρ* = 0.15 m^−1^	0.85237	0.29613	0.49992	0.35157	3.54476
*v_x_* = 10 m/s *ρ* = 0.2 m^−1^	−0.05360	0.12049	0.63999	0.30000	3.02470

**Table 8:** Hardware parameter peaks before and after loop test optimisation under *v_x_* = 15 m/s and *ρ* = 0.15 m^−1^.

	*d_e_* (m)	*r_e_* (rad)	*ω_r_* (rad/s)	*a_y_* (g)	*ϕ* (°)
Before test optimisation	−0.3398	0.4439	0.9504	0.5844	6.0420
After test optimisation	0.7794	0.3256	0.6652	0.4091	4.2326
Percentage of test optimisation	−129.36	26.65	30.01	30.00	29.95
Percentage of simulation optimisation	−130.13	22.28	33.35	33.33	33.29

**Table 9:** Comparative analysis of tracking effect of hardware-in-the-loop test under different speed and different curvature after optimization.

	*d_e_* (m)	*r_e_* (rad)	*ω_r_* (rad/s)	*a_y_* (g)	*ϕ* (°)
*v_x_* = 10 m/s *ρ* = 0.15 m^−1^	−0.01709	0.09224	0.49000	0.22969	2.31667
*v_x_* = 15 m/s *ρ* = 0.15 m^−1^	0.77941	0.32555	0.52498	0.36915	3.72210
*v_x_* = 10 m/s *ρ* = 0.2 m^−1^	−0.05240	0.12049	0.63996	0.30000	3.02378

## Data Availability

The raw/processed data required to reproduce these findings cannot be shared at this time as the data also form part of an ongoing study.
